# High-Fat Diet Aggravates Acute Pancreatitis via TLR4-Mediated Necroptosis and Inflammation in Rats

**DOI:** 10.1155/2020/8172714

**Published:** 2020-01-08

**Authors:** Yu-pu Hong, Jia Yu, Ying-ru Su, Fang-chao Mei, Man Li, Kai-liang Zhao, Liang Zhao, Wen-hong Deng, Chen Chen, Wei-xing Wang

**Affiliations:** ^1^Department of General Surgery, Renmin Hospital of Wuhan University, Wuhan, Hubei, China; ^2^Hubei Key Laboratory of Digestive System Disease, Wuhan, Hubei, China; ^3^Central Laboratory, Renmin Hospital of Wuhan University, Wuhan, Hubei, China

## Abstract

High-fat diet (HFD) often increases oxidative stress and enhances inflammatory status in the body. Toll-like receptor 4 (TLR4) is widely expressed in the pancreatic tissues and plays an important role in pancreatitis. This study is aimed at investigating the effect of HFD on acute pancreatitis (AP) and the role of TLR4-mediated necroptosis and inflammation in this disease. Weight-matched rats were allocated for an 8-week feeding on the standard chow diet (SCD) or HFD, and then, the AP model was induced by infusion of 5% sodium taurocholate into the biliopancreatic duct. Rats were sacrificed at an indicated time point after modeling. Additionally, inhibition of TLR4 signaling by TAK-242 in HFD rats with AP was conducted *in vivo.* The results showed that the levels of serum free fatty acid (FFA) in HFD rats were higher than those in SCD rats. Moreover, HFD rats were more vulnerable to AP injury than SCD rats, as indicated by more serious pathological damage and much higher pancreatic malondialdehyde (MDA) and lipid peroxidation (LPO) levels as well as lower pancreatic superoxide dismutase (SOD) activities and reduced glutathione (GSH) contents and more intense infiltration of MPO-positive neutrophils and CD68-positive macrophages. In addition, HFD markedly increased the expressions of TLR4 and necroptosis marker (RIP3) and aggravated the activation of NF-*κ*B p65 and the expression of TNF-*α* in the pancreas of AP rats at indicated time points. However, TLR4 inhibition significantly attenuated the structural and functional damage of the pancreas induced by AP in HFD rats, as indicated by improvement of the above indexes. Taken together, these findings suggest that HFD exacerbated the extent and severity of AP *via* oxidative stress, inflammatory response, and necroptosis. Inhibition of TLR4 signaling by TAK-242 alleviated oxidative stress and decreased inflammatory reaction and necroptosis, exerting a protective effect during AP in HFD rats.

## 1. Introduction

An increasing number of people are accustomed to sedentary lifestyles and a diet high in fats and refined carbohydrates, and low in fiber, which predispose citizens to the development of many metabolic diseases, including severe obesity, diabetes, and hyperlipidemia. It is well known that diet style is of great importance as a regulative risk factor for oxidative stress in the body [1]. In addition, a high-fat diet (HFD) often results in detrimental metabolic outcomes where oxidative stress is increased by free radical production and an enhanced inflammatory status characterized by higher levels of proinflammatory cytokines [2]. Acute pancreatitis (AP) is an acute inflammatory disorder characterized by autodigestion of pancreatic tissue resulting in local pancreatic injury or systemic inflammatory response [3]. Patients with acute pancreatitis (AP) report experiencing abdominal pain after eating fatty foods, which may often work synergistically with gallstones or alcohol abuse [4]. This indicates the importance of feeding style in the development of AP. Therefore, in this study, we focused on inflammatory organ injury differences in AP between HFD and standard chow diet (SCD) rats.

Toll-like receptors (TLRs), a large family of type I transmembrane proteins, play a critical role in inflammatory response [5]. As the first identified member of the TLR family, TLR4 recognizes a series of exogenous and endogenous ligands, transduces extracellular signal into the cell, and thus mediates inflammation [6]. Besides, TLR4 is widely expressed in the pancreatic tissues, and TLR4 deficiency reduced acinar cell necrosis and attenuated the severity of AP [7, 8]. Also, TLR4 could regulate chemokine formation, neutrophil recruitment, and tissue damage in mice with severe AP [9]. Importantly, necroptosis, a much more aggressive mode of cell death than apoptosis, involved receptor-interacting protein 1 (RIP1), RIP3, and mixed lineage kinase domain-like protein (MLKL) as key molecules [10]. Previously, necroptosis has been described as the predominant mode of acinar cell death in severe experimental pancreatitis [11]. It has been reported that the expressions of RIP1 and RIP3 were negatively related in AP mice [12]. Moreover, emerging evidence indicates that TLR4-induced necroptosis plays an important role in inflammatory diseases [13, 14].

Up to now, little was known about whether TLR4-mediated necroptosis was involved in the development of AP, especially under the condition of HFD. Thus, the present study was designed to investigate the role and mechanism of TLR4-mediated necroptosis and inflammation in AP induced by sodium taurocholate in HFD rats.

## 2. Materials and Methods

### 2.1. Animals

Adult male SPF Sprague-Dawley outbred rats, weighing 200-220 g, were bought from Hunan SJA Laboratory Animal Co. (Changsha, China). The animals were fed standard rodent chow and water, monitored at a controlled temperature, and maintained under a 12 h light/dark cycle for 3 days. The study was approved by the Laboratory Animal Welfare and Ethics Committee of Renmin Hospital of Wuhan University (WDRM-20170505) and performed in compliance with the ARRIVE guidelines and the Guide for the Care and Use of Laboratory Animals from the National Institutes of Health.

### 2.2. Regents

The HFD chow (60% kcal from fat, Research Diets D12492) was purchased from Beijing Huafukang Bioscience Co., and standard chow diet (SCD, 13.2% kcal from fat) was provided by Beijing Keao Xieli Feed Co. Sodium taurocholate (STC) was purchased from Sigma-Aldrich (St. Louis, MO, USA, Cat no. T4009). Transforming growth factor-*β*-activated kinase- (TAK-) 242 was purchased from MedChemExpress (Shanghai, China, Cat no. HY-11109).

### 2.3. Experimental Model and Groups

Following 3-day acclimation, rats were matched with basic body weight and allocated for 8 weeks under different diet treatments. After 8-week feeding on the SCD or HFD, the AP model was induced by a standardized retrograde infusion of 5% STC solution into the rat biliopancreatic duct as previously described [15]. General anesthetic to all rats was induced and maintained by inhaled isoflurane with oxygen during laparotomy.

For determination of the dynamic changes in the inflammatory response and oxidative stress between the HFD and the SCD groups in AP, rats were sacrificed at 3 h, 6 h, and 12 h after the infusion of 5% STC. In a second series of experiments, a potent TLR4 signaling inhibitor, TAK-242 was used *in vivo*. HFD rats were randomly assigned to the following groups: HFD+vehicle group (HV), HFD+AP group (HAP), HFD+AP+TAK-242 (3 mg/kg) group (HAT). TAK-242 were dissolved in 5% DMSO (vehicle) and injected intraperitoneally 30 minutes before AP modeling, and the same volume of vehicle was used in the HV and HAP groups. Rats were anesthetized with isoflurane again and sacrificed 12 h after the infusion of 5% STC. The blood samples were collected from the inferior vena cava and centrifuged for measurement of serum free fatty acid (FFA). The head of pancreas was sampling into 4% phosphate-buffered formaldehyde for histological analysis or immediately frozen in liquid nitrogen for further use.

### 2.4. Oxidative Stress Detection and FFA Measurement

In brief, pancreatic tissues were homogenized in PBS and then centrifuged (12,000 rpm, 4°C, 30 min) to obtain supernatant. The pancreatic level of malondialdehyde (MDA, Cat no. A003-4-1), lipid peroxidation (LPO, Cat no. A106-1-2), reduced glutathione (GSH, Cat no. A006-2-1), and activity of superoxide dismutase (SOD, Cat no. A001-3-2) were analyzed by kits according to the manufacturer's instructions (Nanjing Jiancheng Bioengineering Institute, Nanjing, China). The serum FFA level was measured using standard techniques with a fully automatic chemistry analyzer (ADVIA 2400 Clinical Chemistry System, Siemens Healthcare Diagnostics Inc., New York, USA).

### 2.5. Pancreatic Injury Assessment

According to our previous methods [16], pancreatic tissue samples, fixed in the 4% phosphate buffered formaldehyde, were embedded in paraffin blocks, stained with hematoxylin and eosin, and examined with a light microscope finally. The histopathological scoring analysis of pancreas was performed according to the severity and extent of edema, inflammatory cell infiltration, hemorrhage and acinar necrosis, as described by Schmidt et al. [17]. Six randomly chosen microscopic fields (×200) were examined for each sample, and the extent of acinar cell necrosis was expressed as a percentage of total acinar cells. Cells with swollen cytoplasm, loss of plasma membrane integrity, and leakage of organelles into the interstitium were considered necrotic.

### 2.6. Pancreas Immunostaining

Sections for immunostaining were processed with previous methods [18]. After deparaffinization, hydration, antigen retrieval, and serum blocking, the sections were incubated overnight at 4°C with primary antibodies: MPO (1 : 400, Servicebio, Wuhan, China, Cat no. GB11224), NF-*κ*B p65 (1 : 200, Cell Signaling Technology, Danvers, USA, Cat no. 8242s), and CD68 (1 : 200, Abcam, Cambridge, UK, Cat no. ab12512) and TNF-*α* (Abcam, Cat no. ab6671). Goat anti-rabbit HRP secondary antibody (Maxim Biotech, Fuzhou, China) or Alexa Fluor 488-conjugated secondary antibody (Abcam, Cat no. ab150073) was added to sections at room temperature. Representative images were captured with an Olympus BX63 microscope in light or fluorescent pattern (Olympus, Tokyo, Japan).

### 2.7. Pancreas Western Blotting

Total proteins were extracted using a total protein extraction kit (Beyotime Biotechnology, Shanghai, China), and protein concentrations were determined using the BCA method with bovine serum albumin as a standard. In brief, 20 *μ*g protein samples were separated by 10% SDS-PAGE and then transferred to a polyvinylidene fluoride membrane. The membrane was blocked with 5% skim milk in TBST buffer (TBS containing 0.1% Tween-20) at room temperature for 1 h and then incubated with the following antibodies (1 : 1000 dilution) at 4°C overnight: TLR4 (Servicebio, Cat no. GB11519), RIP3 (Abcam, Cat no. ab56164), and *β*-actin (Servicebio, Cat no. GB11001). After extensive rinsing with TBST, the blots were incubated with secondary goat anti-rabbit (LI-COR, Cat no. 926-32211) antibody (1 : 10000 dilution) at room temperature for 1 h, and the immunoreactive bands were imaged using an LI-COR-Odyssey infrared scanner and Odyssey 3.0 analytical software (LI-COR, Lincoln, USA). The protein bands were quantified by densitometry (Quantity One 4.5.0 software; Bio-Rad Laboratories).

### 2.8. Statistical Analysis

Data were expressed as mean ± standard deviation. The data were analyzed with GraphPad Prism software version 7 (GraphPad Software Inc., San Diego, USA). Statistical significance between two groups was determined by the unpaired two-tailed Student's *t* test and that among multiple groups was determined by one-way or two-way analysis of variance and the Bonferroni post hoc test. Differences were considered statistically significant at a value of *P* < 0.05.

## 3. Results

### 3.1. Dynamic Changes of Histological Injury and Oxidative Stress in the Pancreas of AP Rats

**Figure 1:**
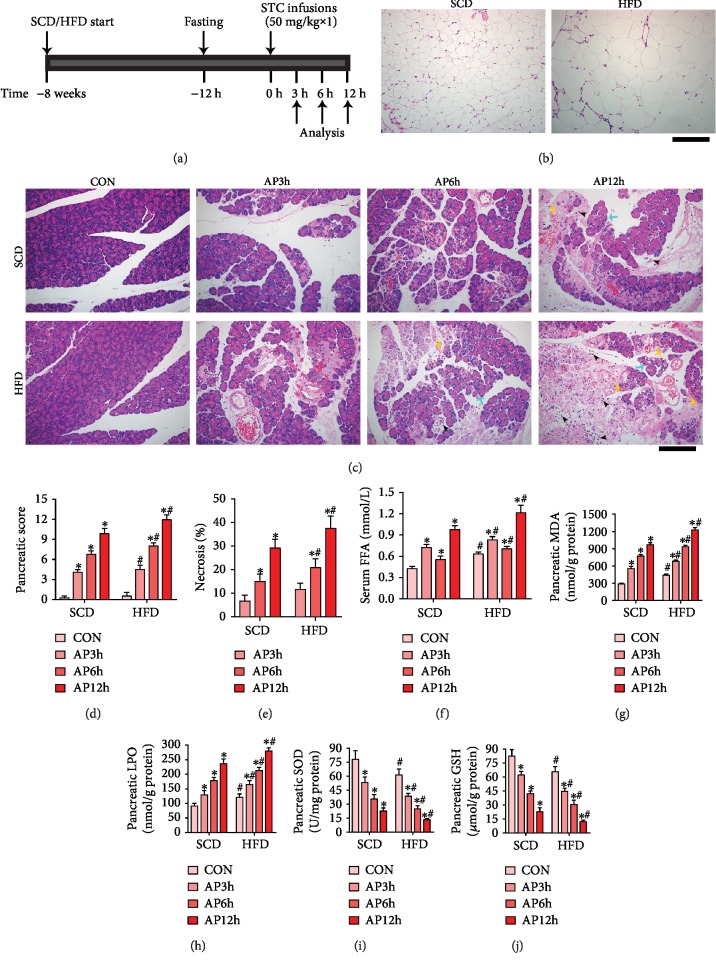
HFD aggravated the histological injury and oxidative stress in the pancreas of acute pancreatitis rats. (a) AP was induced in SCD or HFD rats with a standard retrograde infusion of STC into the biliopancreatic duct; the animals were killed for analysis 3 h, 6 h, and 12 h after the STC infusion. (b) HFD rats showed a larger volume of adipocytes in the peripancreatic fat than SCD rats after an 8-week feeding. (c) In the AP group, serious edema (blue arrow), massive acinar necrosis, inflammatory cell infiltration (black arrowhead), and intrapancreatic hemorrhage (yellow arrow) in the pancreas were observed. (d) Pathological score of pancreatic tissues in rats. (e) Percentage of necrotic cells was determined in the pancreas. (f) Serum levels of free fatty acid in rats. (g) Pancreatic levels of MDA. (h) Pancreatic levels of LPO. (i) Pancreatic activities of SOD. (j) Pancreatic levels of GSH. ^∗^*P* < 0.05 versus prior subgroup, ^**#**^*P* < 0.05 versus SCD group at the same time point. Scale bar = 100 *μ*m.

Pancreatic injury measurements in the biliopancreatic duct infusion model were all made by using portions of the pancreatic head because this model is characterized by changes primarily, and most reproducibly, localized to the head of pancreas [11]. Representative changes of pancreatic tissue are shown in Figure 1. Little morphological evidence of pancreatic injury was observed in the SCD and HFD groups without infusion of STC. However, the volume and size of adipocytes in the peripancreatic fat was larger in HFD-fed rats than those in SCD-fed rats (Figures 1(b)). In AP rats of the SCD group, serious pancreatic edema, massive areas of acinar necrosis, inflammatory cell infiltration, and intrapancreatic hemorrhage were observed in a time-dependent manner. Moreover, the extent and severity of the pancreatic injury and the pancreatic damage scores were significantly increased in AP rats of the HFD group compared with those in AP rats of the SCD group at the same time point (*P* < 0.05, Figures 1(c) and 1(d)). The degree of pancreatic necrosis was elevated after STC infusion in a time-dependent manner and peaked at 12 hours. At each time point, the extent of necrosis in the HFD group was more severe than that in the SCD group (Figure 1(e)).

To investigate the effects of HFD on oxidative stress status, we measured the serum level of FFA; pancreatic level of MDA, LPO, GSH; and activity of SOD in the experimental groups. Compared with the control rats of the SCD group, serum FFA (Figure 1(f)), pancreatic MDA (Figure 1(g)), and LPO (Figure 1(h)) levels were significantly increased in the control rats of the HFD group at baseline, while pancreatic SOD (Figure 1(i)) activity and GSH (Figure 1(j)) content were markedly decreased (*P* < 0.05). In addition, HFD rats subjected to AP showed much higher serum FFA, pancreatic MDA, and LPO levels as well as lower pancreatic SOD activity and GSH content than the SCD rats at the same time points (*P* < 0.05). These results suggest that HFD rats are more vulnerable to AP injury.

### 3.2. HFD Increased the Infiltration of Inflammatory Cell in the Pancreas of AP Rats

**Figure 2:**
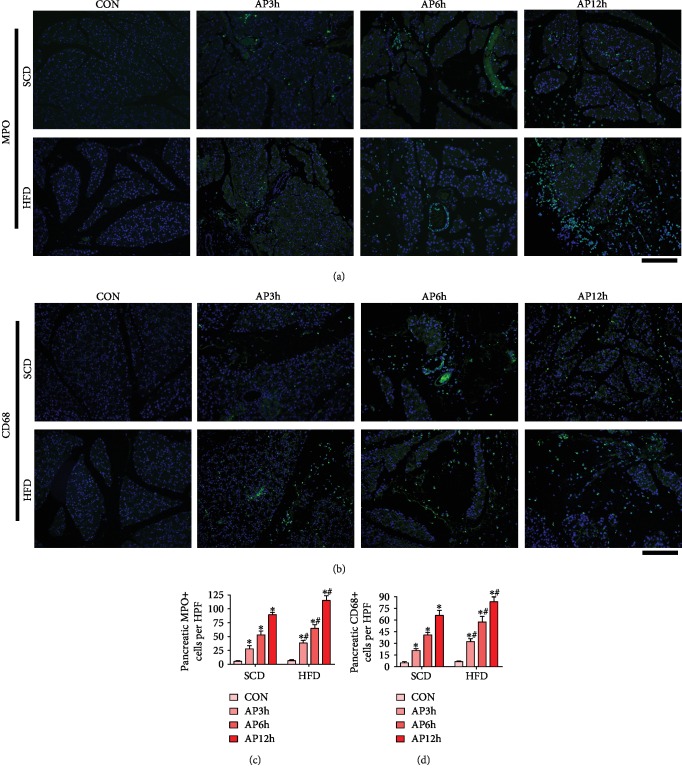
HFD increased the infiltration of inflammatory cell in the pancreas of AP rats. (a) Immunofluorescent staining of MPO-positive neutrophils (green) in the pancreas. (b) Immunofluorescent staining of CD68-positive macrophages (green) in the pancreas. (c) Number of MPO-positive neutrophils. (d) Number of CD68-positive macrophages. ^∗^*P* < 0.05 versus prior subgroup, ^**#**^*P* < 0.05 versus SCD group at the same time point. Scale bar = 100 *μ*m.

As measured by immunostaining (Figure 2), infiltration of inflammatory cell was not significantly different between the control rats of the SCD and HFD groups (*P* > 0.05). During AP, the intense MPO-positive neutrophils and CD68-positive macrophages in the pancreas of HFD rats were increased in a time-dependent manner and were more numerous than in the SCD group at the same time points (*P* < 0.05).

### 3.3. HFD Aggravated Inflammatory Response and Necroptosis in the Pancreas of AP Rats

**Figure 3:**
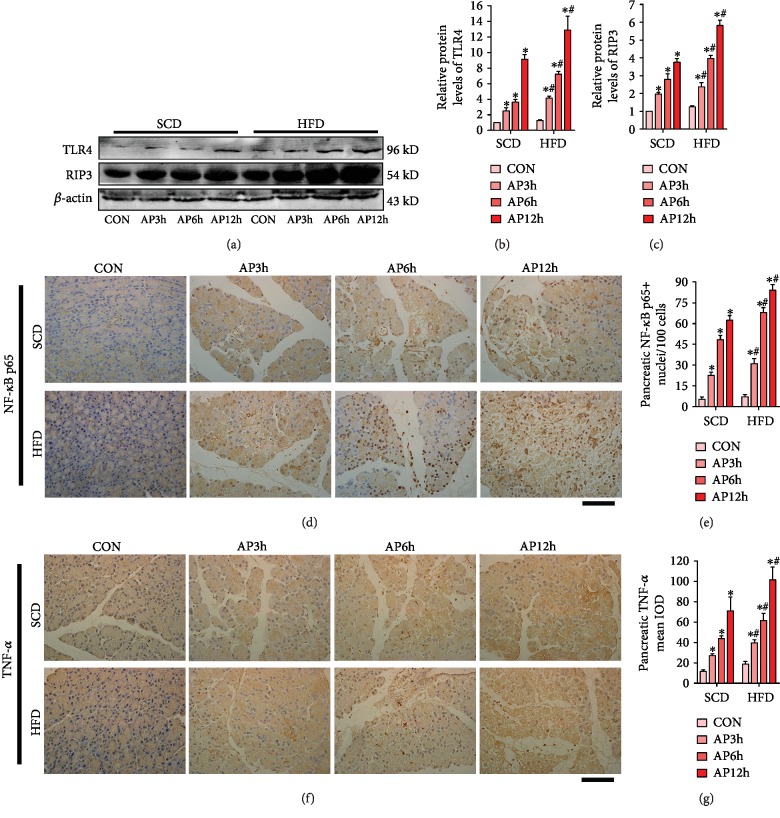
HFD exacerbated inflammatory response and necroptosis in the pancreas of AP rats. (a) TLR4 and RIP3 levels of pancreas were analyzed by Western blot. *β*-Actin served as the loading control. (b) The relative protein expression of TLR4 was expressed as fold changes compared with the control in the blot after normalized to *β*-actin. (c) The relative protein expression of RIP3 was expressed as fold changes compared with the control in the blot after normalized to *β*-actin. (d) Immunohistochemistry of NF-*κ*B p65 in the pancreas. (e) The expression of NF-*κ*B p65-positive nuclei in the pancreas. (f) Immunohistochemistry of TNF-*α* in the pancreas. (g) The mean integrated optical density (IOD) of TNF-*α* in the pancreas. ^∗^*P* < 0.05 versus prior subgroup, ^**#**^*P* < 0.05 versus SCD group at the same time point. Scale bar = 50 *μ*m.

Next, we explored the underlying mechanism of aggravated pancreatic injury in HFD rats. Immunohistochemistry and Western blot experiments were performed. As shown in Figure 3, compared with the AP rats of the SCD group, expressions of TLR4, necroptosis marker (RIP3), expression and nuclear translocation of NF-*κ*B p65, and expression of inflammatory cytokines (TNF-*α*) in the pancreas were noticeably increased in the HFD group as the time goes (*P* < 0.05). However, the above indicators in the pancreas demonstrated no significant changes between the control rats of SCD and HFD groups (*P* > 0.05). These results provided evidence that increased TLR4 expression, inflammatory response, and necroptosis levels may be responsible for aggravated pancreatic injury in AP of HFD rats.

### 3.4. TAK-242 Attenuated Pancreatic Injury and Oxidative Stress of HFD Rats with AP

**Figure 4:**
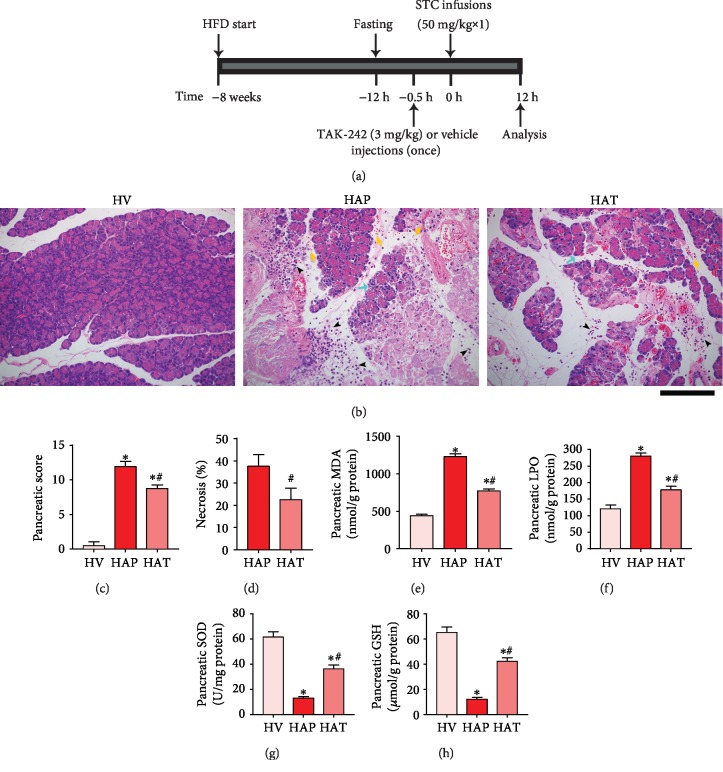
Inhibition of TLR4 by TAK-242 attenuated pancreatic injury and oxidative stress of HFD rats with AP. (a) TAK-242 or vehicle was injected intraperitoneally 30 minutes before AP modeling in HFD rats, and the animals were killed for analysis 12 h after the STC infusion. (b) In the HAP group, serious edema (blue arrow), massive acinar necrosis, inflammatory cell infiltration (black arrowhead), and intrapancreatic hemorrhage (yellow arrow) in the pancreas were observed. TAK-242 improved the histopathological damage. (c) Pathological score of pancreatic tissues in rats. (d) Percentage of necrotic cells was determined in the pancreas. (e) Pancreatic levels of MDA. (f) Pancreatic levels of LPO. (g) Pancreatic activities of SOD. (h) Pancreatic levels of GSH. ^∗^*P* < 0.05 versus HV group, ^**#**^*P* < 0.05 versus HAP group. Scale bar = 100 *μ*m.

We next investigated whether inhibition of the TLR4 signaling pathway with TAK-242 could alleviate AP severity in HFD rats. As shown in Figure 4, compared with the HAP group, treatment with TAK-242 significantly attenuated the extent and severity of the pancreatic injury in the experimental AP rats (Figure 4(b)), and the extent of necrosis and the pancreatic damage scores (Figures 4(c), 4(d)) were significantly decreased as well (*P* < 0.05). Furthermore, TAK-242 reduced AP-induced oxidative stress, as demonstrated by decreased pancreatic MDA (Figure 4(e)) and LPO (Figure 4(f)) levels, as well as increased pancreatic SOD (Figure 4(g)) activity and GSH (Figure 4(h)) content (*P* < 0.05).

### 3.5. Inhibition of TLR4 by TAK-242 Attenuated Inflammatory Response and Necroptosis in the Pancreas of HFD Rats with AP

**Figure 5:**
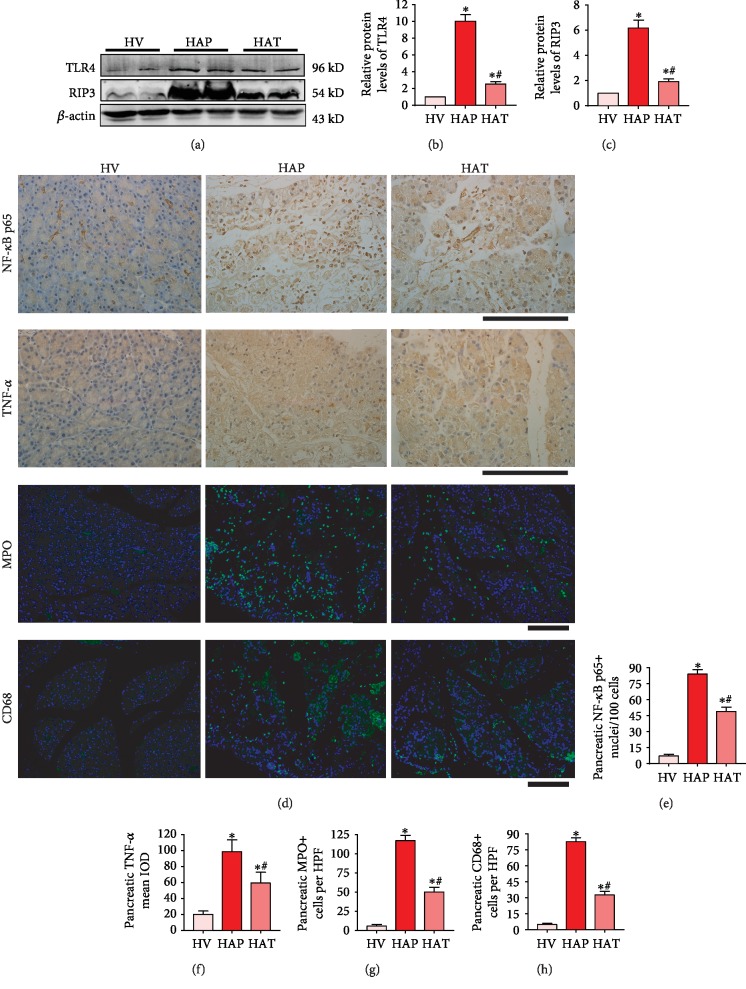
Inhibition of TLR4 attenuated inflammatory response and necroptosis in the pancreas of HFD rats with AP. (a) TLR4 and RIP3 levels of pancreas were analyzed by Western blot. *β*-Actin served as the loading control. (b) The relative protein expression of TLR4 was expressed as fold changes compared with the control in the blot after normalized to *β*-actin. (c) The relative protein expression of RIP3 was expressed as fold changes compared with the control in the blot after normalized to *β*-actin. (d) Immunostaining of NF-*κ*B p65, TNF-*α*, MPO, and CD68 in the pancreas of HFD rats. (e) The expression of NF-*κ*B p65-positive nuclei in the pancreas. (f) The mean IOD of TNF-*α* in the pancreas. (g) Number of MPO-positive neutrophils. (h) Number of CD68-positive macrophages. ^∗^*P* < 0.05 versus HV group, ^**#**^*P* < 0.05 versus HAP group. Scale bar = 100 *μ*m.

As shown in Figure 5, in the HAT group, much lower expressions of TLR4 and necroptosis marker (RIP3), expression and nuclear translocation of p65, expressions of inflammatory cytokines (TNF-*α*) in the pancreas were detected than those in the HAP group (*P* < 0.05). Additionally, inflammatory cell infiltrations consisted of neutrophils and macrophages in the pancreas were significantly decreased in the HAT group, while comparing to the HAP group, (*P* < 0.05). These results collectively suggest that TLR4 inhibition reduces inflammatory response, oxidative stress, and necroptosis in AP of HFD rats.

## 4. Discussion

AP is an acute inflammatory disease of the digestive system. Although the pathogenesis of AP still remains controversial, the oxidative stress pathway is recognized as one of the classical underlying mechanisms in the early phase of AP [19]. Under physiological conditions, oxidative stress damage could be inhibited by antioxidant defense system, consisted of nonenzymatic antioxidants to scavenge reactive oxygen species (ROS) and antioxidase to catalyze the elimination of ROS accumulation [20]. Oxidative stress, caused by an imbalance between oxidant and antioxidant systems, accelerates inflammation by triggering recruitment and activation of inflammatory cells, which in turn aggravates oxidative stress [21]. During AP, excessive production of ROS overwhelming the capacity of antioxidative defense system results in lipid peroxidation of cellular membrane, cytoskeleton disintegration, genetic alterations, and ultimately leading to cell death.

In this study, rats fed with HFD for 8 weeks exhibited increased circulating levels of FFA and caused more severe oxidative stress damage as evidenced by elevated pancreatic MDA and LPO and decreased SOD and GSH, which were in consistent with an earlier research of Yan et al. [22]. In addition, HFD aggravated the severity of AP, as indicated by pathological injury of pancreas, intense infiltration of immune cells, and marked acinar cells necrosis. Previous reports indicate that FFA, especially saturated fatty acid, can activate TLR4-mediated proinflammatory signaling pathways [23, 24]. In accordance with increasing serum FFA levels, the upregulation of TLR4 in the pancreas of HFD rats was also observed during AP. As a result, NF-*κ*B was activated and translocated into nuclei and inflammatory cytokines (TNF-*α*) and RIP3 upregulated in AP rats, which indicated that necroptosis was apparently activated in the inflammatory process of AP, particularly in HFD rats. Accumulating studies indicated that TLR4 activates tissue injury through the RIP3-mediated pathway [25, 26]. In addition, it has been shown that genetic knockout of RIP3 ameliorated the tissue damage of cerulein-induced AP in mice [27]. It has been also reported that chronic HFD administration led to TLR4-mediated RIP3 activation in mice and TLR4 knockdown decreased RIP3 expression and inflammatory response in palmitate-incubated hepatocytes [28]. Moreover, ROS have been considered a driving force for necroptosis, and RIP3-mediated production of ROS positively feeds back on TNF-induced necroptosis [29]. Thus, there may exist a crosstalk between TLR4 and TNF-*α* to form a vicious loop. Furthermore, significant findings have been reported that TLR4-mediated signaling cascades in inflammatory diseases, such as activation of NF-*κ*B and upregulation of TNF-*α* [30]. The activation of NF-*κ*B is critical to the development of AP, and NF-*κ*B probably regulates the onset of AP [31]. Therefore, it was speculated that there may exist a positive feedback loop between RIP3-dependent necroptosis and inflammatory signals, such as TNF-*α* and NF-*κ*B, as a result of inflammatory cascade in AP. Taken together, these results suggested that continuous challenge with HFD can induce fatty acid accumulation and then stimulate TLR4-mediated necroptosis and inflammatory pathways to exacerbate the AP progression.

To confirm the key role of TLR4 in AP of HFD rats, a selective TLR4 inhibitor TAK-242 was used in the present study. TAK-242, also known as resatorvid, is a novel small-molecule compound that binds to the intracellular domain of TLR4 and selectively inhibits TLR4 signal transduction and its downstream signaling cascades and has been widely studied in multiple inflammatory diseases [32]. It has been reported that blocking TLR4 activity via TAK-242 exerts protective effects in pancreatic acinar cells of mice in an *in vitro* AP model [33]. Expectedly, the results of the present study showed that inhibition of TLR4 signal by TAK-242 could downregulate the activation of NF-*κ*B and decrease the expression of RIP3 and TNF-*α*. Prophylactic treatment with TAK-242 also attenuated oxidative stress status by reducing pancreatic MDA and LPO and increasing SOD and GSH. Additionally, TAK-242 diminished migration and infiltration of neutrophils and macrophages in pancreatic tissues of HFD rats with AP. It was gratifying that inhibition of TLR4 exerted a protective effect during AP in HFD rats through lessening the expression of key necroptosis marker, decreasing the inflammatory reaction, and consequently alleviating the injury of pancreas.

## 5. Conclusions

Taken together, the present study demonstrated that HFD could markedly exacerbate pancreatic oxidative stress and inflammatory response and lead to a more notable necroptosis during AP. In agreement with other findings, TLR4 is an important player in the development of FFA-induced inflammatory response and oxidative stress, providing additional evidence for the underlying mechanism of AP process. Inhibition of TLR4 signaling by TAK-242 could reduce oxidative stress and decrease inflammatory reaction and necroptosis, exerting a protective effect during AP in HFD rats. The results of the study could provide a good connection between high-fat diet and TLR4-mediated necroptosis for the pathophysiology of acute pancreatitis.

## Data Availability

The data used to support the findings of this study are included within the article.
